# Synthesis and evaluation of nanosystem containing chondroitinase ABCI based on hydroxyapatite

**DOI:** 10.1186/s13568-024-01677-5

**Published:** 2024-02-14

**Authors:** Fatemeh Afraei, Sara Daneshjou, Bahareh Dabirmanesh

**Affiliations:** 1 Department of Nanobiotechnology, Faculty of Biological Science, Tarbiat Modares University, Tehran, Iran; 2 Department of Biochemistry, Faculty of Biological Science, Tarbiat Modares University, Tehran, Iran

**Keywords:** *Chondroitinase ABCI*, Drug delivery, Hydroxyapatite, Nanosystem

## Abstract

The bacterial enzyme chondroitinase ABCI (chABCI), which has been isolated from *Proteus Vulgaris*, is crucial in the treatment of spinal cord injuries. However, due to its short lifespan, the maintenance and clinical application of this enzyme are very constrained. In this study, the immobilization of this enzyme on hydroxyapatite has been carried out and assessed with the aim of enhancing the characteristics and efficiency of chABCI. Hydroxyapatite particles (HAPs) are a potential candidate for drug-delivery carriers because of their excellent biocompatibility, shape controllability, and high adsorption. The use of the nanometer scale allows efficient access to the enzyme's substrate. It demonstrates important biological application capabilities in this way. Field emission gun-scanning electron microscopy (FEG-SEM), X-ray diffraction (XRD), infrared spectroscopy (FT-IR), in vitro release study, and cytotoxicity test were used to characterize the drug nanosystem's properties. According to the findings, electrostatic bindings was formed between charged groups of the enzyme and hydroxyapatite nanoparticles. The results also demonstrated that immobilized chABCI on hydroxyapatite has beneficial properties, such as more manageable drug release, minimal toxicity and side effects, and a high potential to enhance the efficacy of drug delivery and decrease the need for repeated injections.

## Key points

- It has been proven that hydroxyapatite increases the shelf life of chondroitinase when it is used as a carrier matrix.
- Stability of the enzyme loaded onto hydroxyapatite was enhanced significantly after immobilization.
- The proposed process can make the hydroxyapatite nanoparticles a useful stabilizer drug delivery vehicle.
- The release of the chondroitinase is more controllable than previous studies; therefore the stability of the nanosystem has been increased.

## Introduction

The development of a glial scar is one of the several reactive alterations that take place after spinal cord damage (Bradbury et al. 2011; Fawcett et al. 1999; Daneshjou et al. 2017). Glial cells gather and encircle the location of CNS damage during this reactive cellular process to close the wound. By restoring the CNS's physical and chemical integrity, sealing the blood-brain barrier, limiting the invasion of non-CNS tissue, and limiting the spread of infection and cellular damage, glial scar formation plays a critical role in preserving CNS tissue. Axonal projections cannot develop or be repaired because the glial scar acts as a physical and chemical barrier to the regeneration of injured axons (Bradbury et al. 2011; Faulkner et al. 2004). Although chemical elements found in the extracellular matrix of the glial scar may actively limit axon extension, the physical presence of a dense and reactive glial cell hinders growth cone advancement (Bradbury et al. 2011; Busch et al. 2007). Chondroitin sulfate proteoglycans (CSPGs) are inhibitory molecules connected to the extracellular matrix of the glial scar (Bradbury et al. 2011; Muir et al. 2019). A protein core with one or more covalently linked glycosaminoglycan-chondroitin sulfate (GAG-CS) chains makes up chondroitin sulfate proteoglycans (Bradbury et al. 2011). Studies on the bacterial enzyme chondroitinase ABCI led to the discovery that the primary inhibitory component of the chondroitin sulfate proteoglycan (CSPG) molecules are the sugar chains of glycosaminoglycans (GAGs). The proteoglycans of chondroitin sulfate are broken down into soluble disaccharides or tetrasaccharides by the bacterial enzyme chondroitin sulfate ABC lyase (cABC1, EC 4.2.2.4), which was isolated from the bacterium *Proteus Vulgaris* (Bradbury et al. 2011; Muir et al. 2019). This reduces the inhibitory effect of proteoglycans of chondroitin sulfate. The therapeutic benefits of chondroitinase ABC were first established as a method to encourage nerve regeneration in early research. By intrathecal injection, the enzyme was delivered to the spinal cord. Nevertheless, since the enzyme rapidly loses its activity, this procedure could need numerous injections to be successful. Other methods of enzyme administration were thus investigated. At 37 °C, chondroitinase ABC is unstable, and the majority of its activity is lost in about 72 h. To improve the enzyme stability and ensure its gradual release, the immobilization of ABC chondroitinase on different materials has been investigated (Daneshjou et al. 2017; Nazari et al. 2018; Muir et al. 2019). Using poly(propylene carbonate)-chitosan microfibres to delay chondroitinase ABC release, an in vitro study revealed that the majority of the enzyme was released for up to 10 days, but that only 26% of this quantity was an active enzyme. The findings indicate that this distribution strategy presently has no appreciable impact on performance enhancement. Nanoparticles distribute chondroitinase ABCI more effectively than microfibers made of poly(propylene carbonate) and chitosan (Muir et al. 2019). The spinal cord requires a broad variety of scientific and healing capabilities, and nanoparticles are potent tools that can do these tasks. They also hold promise for delivering medications to the injured spinal cord (Zuidema et al. 2016). In a study by Daneshjou et al., the stability of the enzyme was significantly increased at various temperatures by using porous silicon nanoparticles as a stabilizing substrate for transporting chABCI and stabilizing the enzyme. For instance, the immobilized enzyme preserved 50% of its original activity after 100 min at 4 °C, whereas the free enzyme only retained 20% of the activity (in 10 mM imidazole) (Daneshjou et al. 2017). The enzyme is shielded in the pores of porous silicon nanoparticles, which increases stability, according to different researches done by the same team (Daneshjou et al. 2020). Several strategies for enzyme immobilization have been introduced as a result of recent developments in nanotechnology and nanomaterials (Daneshjou et al. 2017; Bosio et al. 2016). Drug-loading research has concentrated on the creation of particle medication delivery methods over the last 10 years. Due to their biocompatibility and bioaffinity, as well as their good biological activity and great stability, bioceramic particles have garnered increased attention among these systems (Wen et al. 2021). The majority of human bones and teeth are made of hydroxyapatite, which has the chemical formula (Ca_10_)(PO_4_)_6_(OH)_2_. Moreover, its bioactivity, biodegradability, and osteoconductive characteristics have been researched as essential biocompatible materials owing to its chemical resemblance to the mineral calcium phosphate present in hard biological tissues (Moselemi et al. 2015; Bellucci et al. 2019). They are a great option for drug carriers as a result. In general, hydroxyapatite has a lengthy biodegradation period and may be maintained with continuous drug release long after injection. As a result, drug release can be tightly controlled for a long time, and complications from repeated injections and side effects from high doses can be avoided. The P site has a negative charge while the Ca site has a positive charge in the hydroxyapatite crystal structure, which is hexagonal. Because of these characteristics, hydroxyapatite functions well as an adsorbent and can bind to a variety of substances, including proteins, antitoxins, and growth factors (Wen et al. 2021). Nano-hydroxyapatite has been used as a drug carrier, according to earlier studies. The anti-inflammatory drug ibuprofen, the antibiotics norfloxacin and vancomycin, the bisphosphonate alendronate, the cardiovascular drug carvedilol, the anticancer drug cis-diamminedichloroplatinum (II), the di(ethylenediamineplatinum) medronate, and others have all been carried by nano-hydroxyapatite (Nur Farahiyah et al. 2014). Additionally, nano-hydroxyapatite was used to effectively stabilize these enzymes with a straightforward uptake protocol while maintaining their catalytic activity for the immobilization of β-glucosidase, protease, and phytase with industrial applications as well as dextranase, which is involved in the breakdown of dental plaque (Coutinho et al. 2018; Zdarta et al. 2015; Coutinho et al. 2020; Ding et al. 2020). A different study used the hydroxyapatite drug composition to release amoxicillin to treat bone infections (Prasanna et al. 2018). The current study was conducted to evaluate the stability and toxicity of free and immobilized enzyme on hydroxyapatite. First, X-ray diffraction and field emission gun-scanning electron microscopy (FEG-SEM) were used to characterize nano-hydroxyapatite. UV spectroscopy was used to assess the enzyme loading and release capabilities of hydroxyapatite nanoparticles. Additionally, (FEG-SEM) imaging, XRD, and FT-IR were used to characterize the immobilized enzyme on hydroxyapatite. The findings demonstrated that nano-hydroxyapatite can be regarded as a carrier with a high potential for stabilizing chondroitinase ABCI.

## Materials and methods

### Materials

Chemicals including chondroitin 4-sulfate, potassium dihydrogen phosphate (KH_2_PO_4_), and kanamycin were all purchased from Sigma Aldrich; (USA), and nickel NTA agarose was purchased from Qiagen (USA). Isopropyl β-d-1-thiogalactopyranoside (IPTG) was prepared from Takara (Japan). The nano-hydroxyapatite powder was obtained from Fine Nano company (Iran, Tehran). Deionized water and phosphate buffer were used to make the solutions.

### Enzyme expression and purification

Competent BL21 cells were transformed using the pET-28a plasmid containing wild-type cABC (GenBank^®^ accession number E08025) (Prabhakar et al. 2005). The transformant were grown overnight at 37 °C. The following morning, the overnight culture was used to inoculate 500 ml of Luria–Bertani (LB) medium with kanamycin (0.05 mg/ml). 0.7 mM IPTG was used to induce the cultures for 6 h at 27 °C. The cells were then separated by centrifugation (3500*g*, 10 min, 4 °C) to obtain a cell pellet. Subsequently, the pellet was re-suspended in lysis buffer (50 mM potassium phosphate, 300 mM NaCl, 5 mM imidazole, and 1 mM phenylmethanesulfonyl fluoride (PMSF); pH 6.8) and sonicated to lyse the cells. To remove the cell lysate, the sonicated mixture was centrifuged at 15,500*g* for 20 min. the supernatant was loaded onto A Ni–NTA column equilibrated with 50 mM phosphate buffer at pH 6.8. The enzyme was purified using gradient of 0-300mM imidazole (Daneshjou et al. 2017; Daneshjou et al. 2020; Nazari-Robati et al. 2013; Naderi et al. 2018). The SDS-single PAGE band was used to evaluate the protein’s purity. Bradford's method was used to estimate the protein concentration (Daneshjou et al. 2017; Daneshjou et al. 2020; Nazari-Robati et al. 2013; Bradford 1976).

### Immobilization procedure

To immobilize the enzyme onto hydroxyapatite nanoparticles, 0.1 mg of nano-hydroxyapatite powder was added to 0.2 ml of chABCI enzyme at a concentration of 0.2 mg/ml. The mixture was sonicated for 75 min to completely disperse the particles in the enzyme solutions (Askaripour et al. 2019). Then, the mixture stirred gently for 1, 4, and 12 h at 4 °C (Coutinho et al. 2018; Zdarta et al. 2015; Coutinho et al. 2020). Then centrifugation was carried out (20,000*g*, 20 min, 4 °C). The remaining precipitate (immobilized enzyme) was then dissolved in 200 µl phosphate buffer 50 mM.

### Activity measurement of immobilized chABCI enzyme

UV-Vis spectrophotometry was used to measure the by-product formation of immobilized chABCI activity. In this regard, it was determined how much the absorbance increased over time in phosphate buffer (pH 6.8, 25 °C) as a function of wavelength at 232 nm. 40 μl of immobilized enzyme (with 4 μg enzyme in this volume) was added to 250 µl of 50 mM phosphate buffer (pH 6.8) which contains concentration of chondroitin-4-sulfate (C4S, 1 mg/ml). For the calculations, the molar absorption coefficient (ꜫ) of 3800 M^−1^ cm^−1^ was used. The amount of enzyme that releases 1 µmol of unsaturated oligosaccharides per minute under the prescribed assay conditions is referred to as one unit of chABCI activity (Daneshjou et al. 2017; Daneshjou et al. 2020; Nazari-Robati et al. 2013; Askaripour et al. 2019; Yamagata et al. 1968).

### Thermal stability of the nanosystem (Immobilized chABCI on hydroxyapatite nanoparticles) and free enzyme

The immobilized and free enzyme were stored at 25 °C and 37 °C and the stability and residual activity at both temperatures were examined at various time intervals (Daneshjou et al. 2020).

### In vitro release

After being incubated at 25 °C for various times with 0.1 mg of nano-hydroxyapatite powder was incubated with the immobilized enzyme (0.1 mg/ml) in phosphate buffer (pH 6.8) and centrifuged, the protein release was detected in the supernatant at 220 nm (Daneshjou et al. 2020).

### Characterization methods

The morphology of hydroxyapatite nanoparticles before and after enzyme immobilization was investigated using field emission gun-scanning electron microscopy (FEG-SEM) (Zeiss, Germany). The crystallinity of hydroxyapatite nanoparticles was measured by X-ray diffraction using an X Pert Pro XRD device (Panalytical). The diffraction data were collected in the range of 2θ = 5–80°. Infrared spectroscopy (Ft-IR) was used to analyze hydroxyapatite nanoparticles before and after ABCI immobilization using a Nicolet IR100 FT-IR (Termo Scientific); Transmission measurements were performed in the mid-infrared range of 400–4000 cm^−1^.

### MTT assay

In order to determine cytotoxicity, cell viability was assessed using a 3-(4,5-dimethylthiozol-2-yl)-2,5-diphenyltetrazolium bromide (MTT) assay. Selected PC12 cell lines (from Iran’s Pasteur Institute) were cultured in 96-well plates. The seeded plates were cultured for 24 h in a humid incubator at 37 °C and 5% CO_2_. 100 ml of test compounds (free nano-hydroxyapatite and immobilized enzyme on hydroxyapatite nanoparticles) and culture medium were applied to the cells in each well after the culture media had been removed. For the free hydroxyapatite nanoparticles and the immobilized enzyme, concentrations of 20 µg/ml, 50 µg/ml, 100 µg/ml, 200 µg/ml, and 10 µg/ml, 20 µg/ml, 40 µg/ml, 80 µg/ml, 120 µg/ml, and 160 µg/ml, respectively, were utilized. Wells that solely contained growth media (without the test chemical) were used as controls. Samples were examined after being stored for 12, 24, and 48 h. When the requisite amount of time had passed, the plates were taken out of the incubator, a quantity of MTT solution equal to one-tenth of the supernatant was added to each well, and the plates were then returned to the incubator for an additional 4 h. The purple formazan crystals in the live cells were then dissolved, and a homogenous chromogenic liquid was created by draining the supernatant and replacing it with DMSO after the plate had been removed. The absorbance was measured at 570 nm when the plates were transferred to an ELISA plate reader (using a 620 reference filter). Cell vitality (%) = the absorbance of the experimental group/the absorbance of the control group in white × 100%) was calculated by dividing the sample's absorbance by the absorbance of the identical controls as the time point (Daneshjou et al. 2020).

## Results

### Immobilization of chABCI on hydroxyapatite

**Fig. 1:**
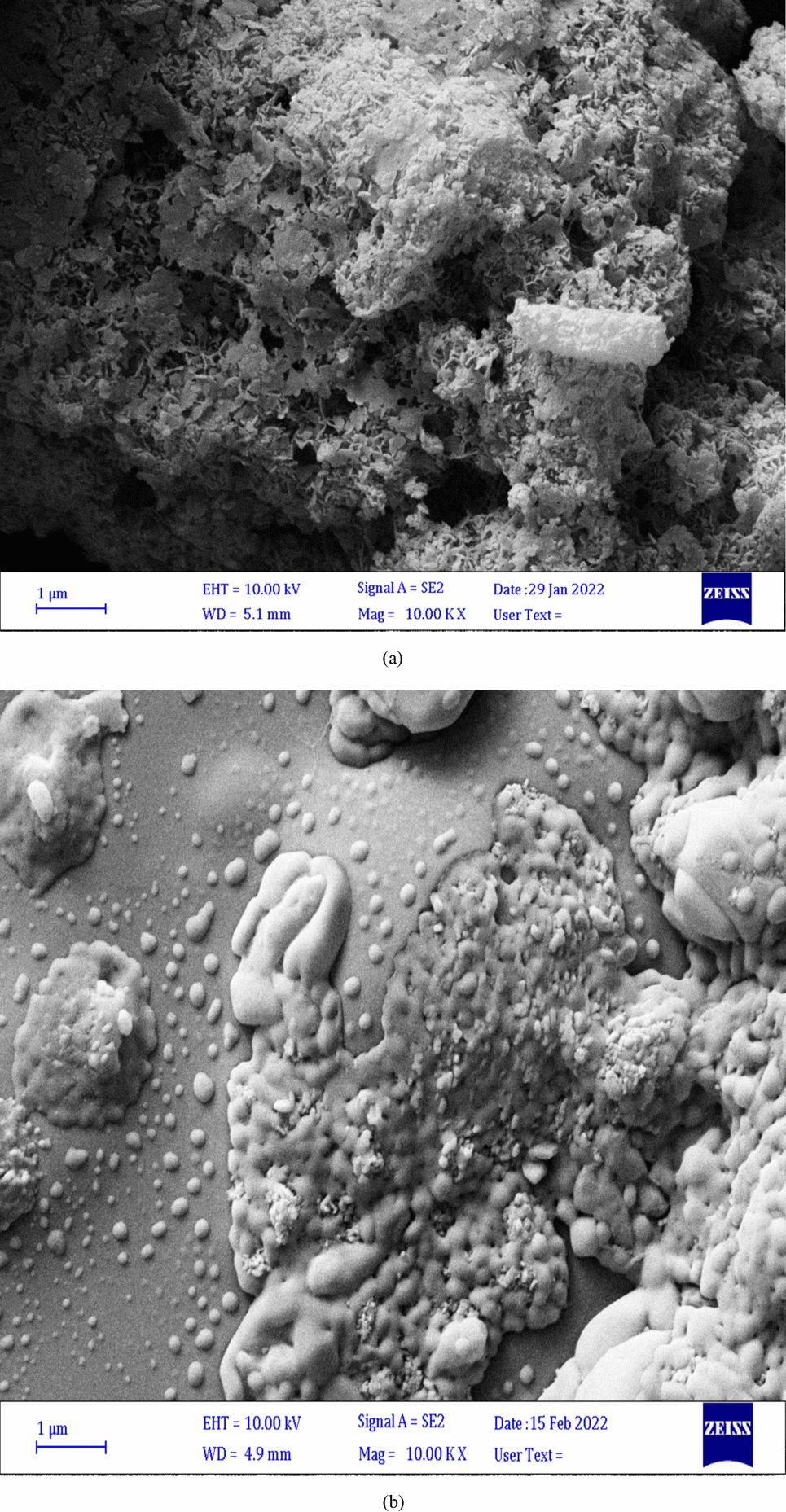
**a** SEM image of virgin hydroxyapatite nanoparticles. **b** SEM image of immobilized chondroitinase on hydroxyapatite nanoparticles

cABC I was expressed in *E. coli* BL21 and SDS-PAGE was used to determine its purity as described by Daneshjou et al. 2020. In the following, the nano-hydroxyapatite powder was used to immobilize the enzyme. Dimensions of chABCI were obtained (a = 49.28 (Å), b = 95.14 (Å), and c = 229.5 (Å)) from the enzyme crystallography data (PDB No: 1HN0) (Askaripour et al. 2019). The cations exposed (Ca^2+^) on the inorganic matrix can establish ionic adsorption with negatively charged proteins, and the carboxylic acid side chains of Asp and Glu residues abundant on the chABCI surface (Coutinho et al. 2018). It was hypothesized that calcium ions would chelate the carboxylic acid groups of the aspartate (Asp) and glutamate (Glu) residues in the amino acid side chains owing to the presence of aspartate and glutamate in the structure of the enzyme. As a consequence, the approach of chABCI's direct contact with nanoparticles was used to carry out the immobilization procedure. Immobilization was carried out for 1, 4, and 12 h (During the incubating process of the enzyme with hydroxyapatite, the buffer contained imidazole, as previouly reported by Daneshjou et al. (2017). Because the enzyme activity remains constant in the presence of imidazole). Based on Coutinho et al. (2018), Coutinho et al. (2020), Ding et al. (2020), Qi et al. (2020), Daneshjou et al. (2017) studies, each sample is containing 0.1 mg of hydroxyapatite and 200 µl of the enzyme at a concentration of 0.2 mg/ml. In the following, after determining the best incubation time to immobilize the enzyme. A variety of protein concentrations were tested to determine the optimal concentration for immobilization. Up to the concentration of 0.2 mg/ml of enzyme, the activity increased, but no changes in the activity were observed as the concentration increased. At first, the enzyme and nano-hydroxyapatite powder combination was sonicated to ensure that all of the nanoparticles were evenly distributed throughout the enzyme solution. The samples were then kept at 4 °C on the stirrer at the optimal incubation time. Subsequently, they were centrifuged at 20,000×*g* for 20 min. SEM was used to establish the chABCI immobilization on hydroxyapatite nanoparticles (Daneshjou et al. 2020). The SEM picture of the immobilized chABCI on nano-hydroxyapatite is shown in Fig. 1b. As shown in the SEM picture, the surface of the enzyme seems to be coated with a layer after immobilization which was similar to that observed in the article by Daneshjou et al. (2020). Based on the given equation the efficiency of the enzyme immobilization was estimated to be 41.6%. The protein content of enzyme solutions was determined according to the Bradford method.

$$\frac{(\mathrm{Initial \,concentration \,of \,enzyme},\mu g)-(\mathrm{Concentration \,of \,free \,enzyme}({\text{supernatant}} ),\mu g)}{\mathrm{Initial \,concentration \,of \,enzyme} (\mu g)}\times 100$$

$$=\frac{(48-28)}{48}\times 100=41.6\%.$$

According to the mentioned cases, about 20 µg of enzyme was immobilized on 0.1 mg of hydroxyapatite, which was dissolved in 200 µl phosphate buffer 50 mM to continue the work.

### Characterization of chABCI immobilized on hydroxyapatite

**Fig. 2:**
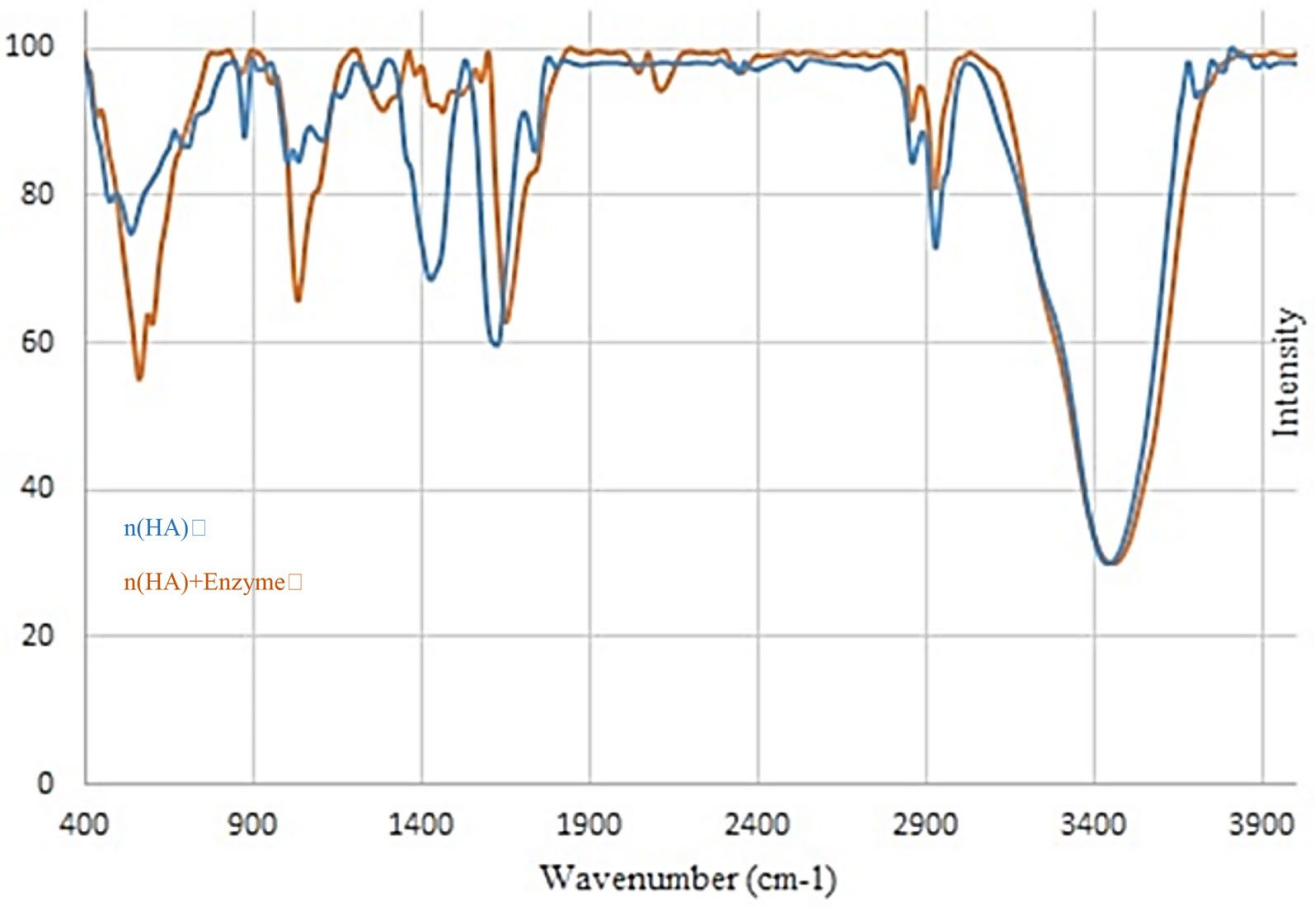
FT-IR spectra of hydroxyapatite (HA) with and without immobilized chondroitinase; Comparison of the hydroxyapatite spectrum before and after enzyme immobilization shows changes in the peaks of the functional groups. The obtained FT-IR spectrum confirms the immobilization of the enzyme on the surface of the nanoparticles

**Fig. 3:**
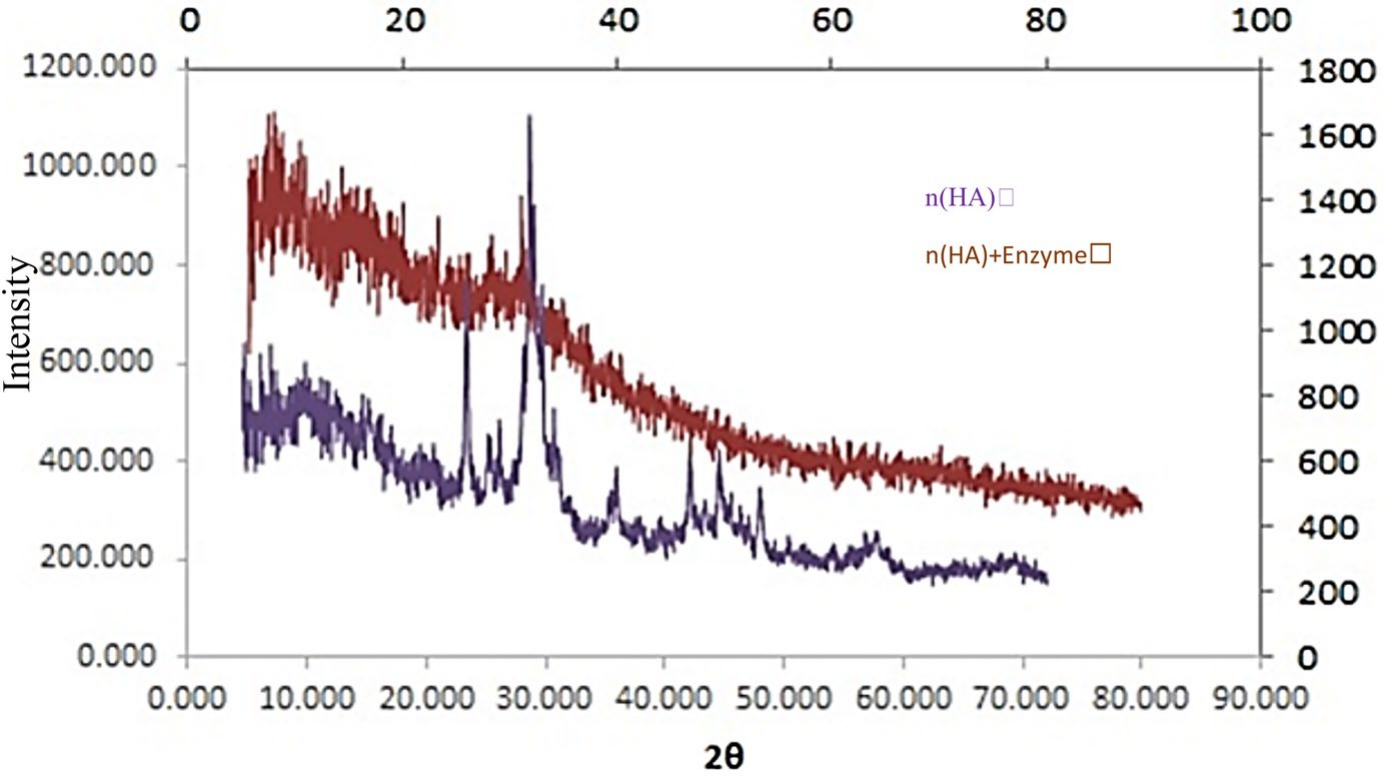
X-ray diffractogram of the hydroxyapatite support before and after of ABCI chondroitinase immobilization

The surface morphology of nanoparticles was investigated using the field emission gun-scanning electron microscopy (FEG-SEM) (Fig. 1). The hydroxyapatite nanoparticles utilized in this work had a typical size of 25 nm and were needle-shaped. Scanning electron microscopy analysis of the hydroxyapatite nanoparticles provided proof that chondroitinase was immobilized (Daneshjou et al. 2020). The surface characteristics of bare nano-hydroxyapatite are shown in Fig. 1a using a scanning electron micrograph. The SEM picture of hydroxyapatite after chondroitinase immobilization is shown in Fig. 1b. It is evident that an enzyme layer nearly completely covers the surface. The layers of the enzyme seem to cover the surface. FT-IR spectroscopy was used to look into the existence of surface functional groups and the binding of chondroitinase to hydroxyapatite. Figure 2 shows the spectra for hydroxyapatite with and without immobilized chondroitinase. The absorption bands represent the hydroxyl and phosphate groups found in the hydroxyapatite (Ca_10_)(PO_4_)_6_(OH)_2_ molecules. The OH groups of hydroxyapatite or the vibration of water molecules adsorbed on the sample's surface may be responsible for the bands at 3572 and 3445 cm^−1^ (Coutinho et al. 2020; Wu et al. 2010). Due to the hydroxyl groups of amino acid residues like serine, threonine, and tyrosine, both of these bands intensified in the presence of enzyme (Coutinho et al. 2020; Kumar et al. 2016). The stretching of –CH linked to the side chains of amino acid residues, such as the alkane side chain of lysine, might be the cause of the vibrational bands seen at 2953, 2934, and 2869 cm^−1^ in the spectra for hydroxyapatite with enzyme (Fig. 2). Bands at 1650, 1421, and 1458 cm^−1^ are seen in the spectra for both types of hydroxyapatite support (with and without enzyme). These bands, which are C=O stretching vibrations, might point to the existence of a second CaCO_3_ phase or the absorption of ambient CO_2_ by hydroxyapatite nanoparticles (Coutinho et al. 2020). Since amide functional amino acid residues like asparagine and glutamine have a C=O stretching vibration, the spectra for the support with immobilized enzyme exhibited a larger intensity at 1650 cm^−1^. Moreover, the carboxylic acids of amino acid residues like aspartate and glutamate were the cause of the stronger band intensities at 1421 and 1458 cm^−1^. These results show that chondroitinase was successfully immobilized onto the hydroxyapatite nanoparticles. A specific band at 1549 cm^−1^ in the spectrum for hydroxyapatite with immobilized chondroitinase could be attributed to absorbance by the amino groups of the enzyme, as previously observed in the immobilization of β -glucosidase on hydroxyapatite nanoparticles (Coutinho et al. 2018; Coutinho et al. 2020). Also, the crystal phases contained in the hydroxyapatite structure were found using the X-diffraction pattern (XRD) (Fig. 3). According to XRD studies, HA's crystal shape is unaltered following immobilization. The hydroxyapatite peaks were made stronger by the addition of chondroitinase, and the relative intensities of each peak are in excellent accord with the hydroxyapatite X-ray diffraction pattern (Fig. 3) (Ding et al. 2020).

### Activity measurement of immobilized chABCI enzyme

**Fig. 4:**
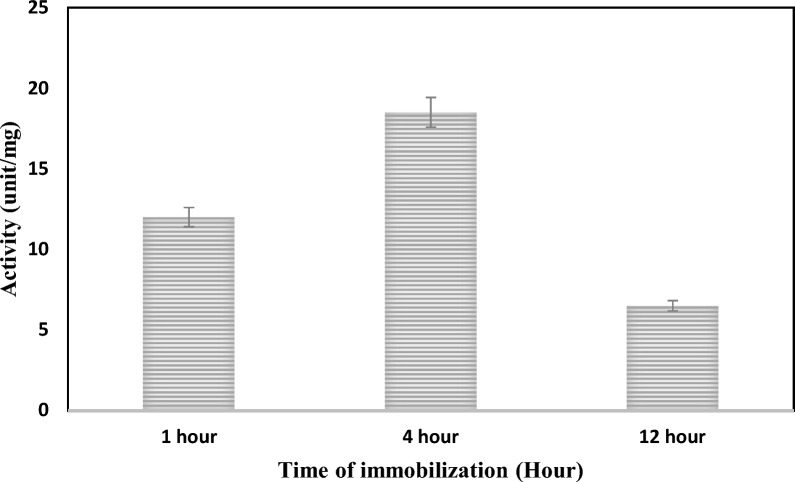
The activity of immobilized enzymes in 1 h, 4 h, and 12 h of incubation. The results indicated that the immobilized enzyme in 4 h had more activity than the immobilized enzymes in 1 h and 12 h

On the basis of product production, the immobilized chABCI was assessed using UV-vis spectrophotometric technique. The immobilized enzymes’ activity at 1, 4, and 12 h is shown in Fig. 4. The highest activity was seen at 4 h and reduced as the incubation time increased. Therefore, 4 h was chosen for chABCI immobilization in our experiments.According to Prasanna et al. (2018) and Konwarh et al. (2009), studies, the enzyme could loose its activity after 12 h. They reported that as the immobilization time increases, the enzyme can migrate to the bottom of the support and is no longer able to bind to the substrate. Furthermore, we also assumed that the loss of activity could be due to the reduction in the enzyme stability after 12 h of incubation.

### Thermal stability of the nanosystem (Immobilized chABCI on hydroxyapatite nanoparticles) and free enzyme

**Fig. 5:**
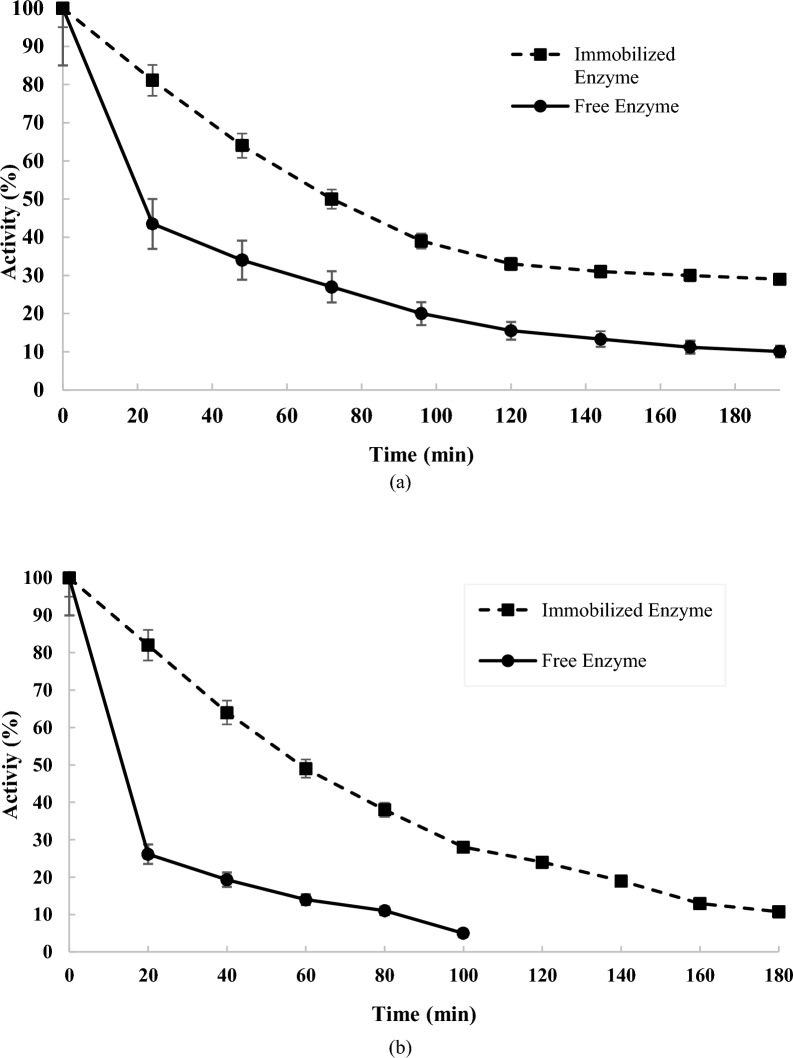
Thermal stability of the free and immobilized chABCI at (**a**) 25 °C (**b**) 37 °C in 50 mM phosphate buffer (pH 6.8). Standard deviations were within 6% of the experimental values

By incubating the free and immobilized enzyme at 25 °C and 37 °C, the stability of the free and immobilized chABCI was examined. The results are shown in Fig. 5. According to the findings, immobilized chondroitinase was more stable than free chondroitinase. After 100 min at 25 °C, the immobilized enzyme had preserved 40% of its original activity, while the free enzyme had retained around 20%. After incubation at 25 °C, the immobilized enzyme's activity remained constant for two weeks (Fig. 5a). Moreover, the nanosystem's stability at 37 °C was assessed. The nanosystem kept around 35% of its original activity at 37 °C after 100 min, while the free enzyme lost its activity after that time (Fig. 5b). As the immobilized enzyme is intended for usage in the body, the rise in the nanosystem's stability at 37 °C might serve as a favorable sign for enhancing the clinical applicability. The enzyme's conformational modifications and the chABCI slipping off the carrier's surface may both be to blame for the immobilized chABCI's activity decrease (Danping et al. 2020). The effective immobilization of β-glucosidase, dextranase, protease, and phytase on nano-hydroxyapatite has previously been described (Coutinho et al. 2018; Coutinho et al. 2020; Ding et al. 2020; Danping et al. 2020). Nano-hydroxyapatite's breakdown product is far less hazardous than those of silica, quantum dots, carbon nanotubes, and magnetic particles. By replacing different kinds of ions, such as carbonate, chloride, or fluoride, hydroxyapatite's dissolution may be regulated, and thanks to the material's solubility, chemicals or medications can be released in certain target locations (Nur Farahiyah et al. 2014).

### In vitro release

**Fig. 6:**
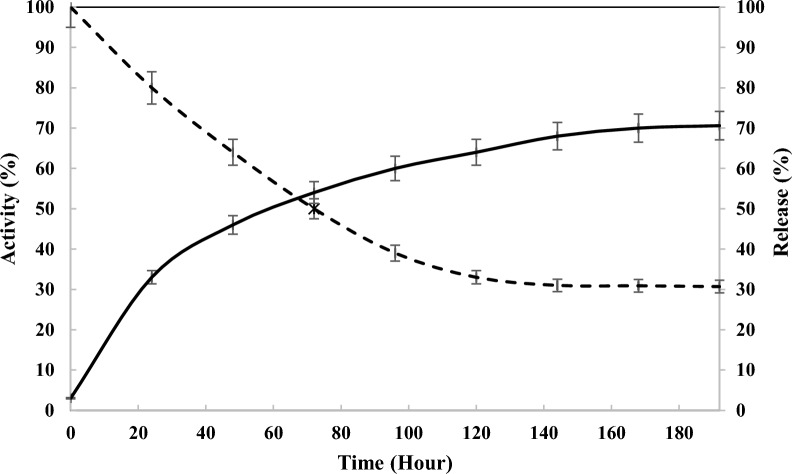
Percentages of chondroitinase activity (—) and release (—) in phosphate buffer at 25 °C as a function of time. Standard deviations were within 5% of experimental values

At various intervals, the immobilized enzyme (in phosphate buffer) was centrifuged. The absorbance of the phosphate buffer (pH 6.8) containing the released chABCI at 220 nm (the absorption of peptide bonds was measured at this wavelength) was used to calculate the quantity of enzyme released from hydroxyapatite nanoparticles (Nazari-Robati et al. 2013).the absorbance of the supernatant rose with time at 220 nm (protein content) to ensure protein release. (Naderi et al. 2018). The six-day drug release evaluation period. as shown in Fig. 6.around 54% of the enzyme is released after 3 days; also, enzyme activity was measured at 232 nm. according to (Fig. 6) the activity of the enzyme reduced at 232 nm which is due to the release of the enzyme from hydroxyapatite.after 6 days, the activity falls to 31%, and this level is maintained for 2 weeks. Due to the drug's poor interaction with the hydroxyapatite particles, the release of medicines from hydroxyapatite was shown to be initially quite quick (Prasanna et al. 2018; Mizushima et al. 2006). The hydroxyapatite releases drugs at a high rate at first, then decreases. The hydroxyapatite shrinking causes a sharp rise in drug concentration. The surface-located chABCI molecules are promptly released. The concentration of the medication rises quickly as a result (Prasanna et al. 2018).

### MTT assay

**Fig. 7:**
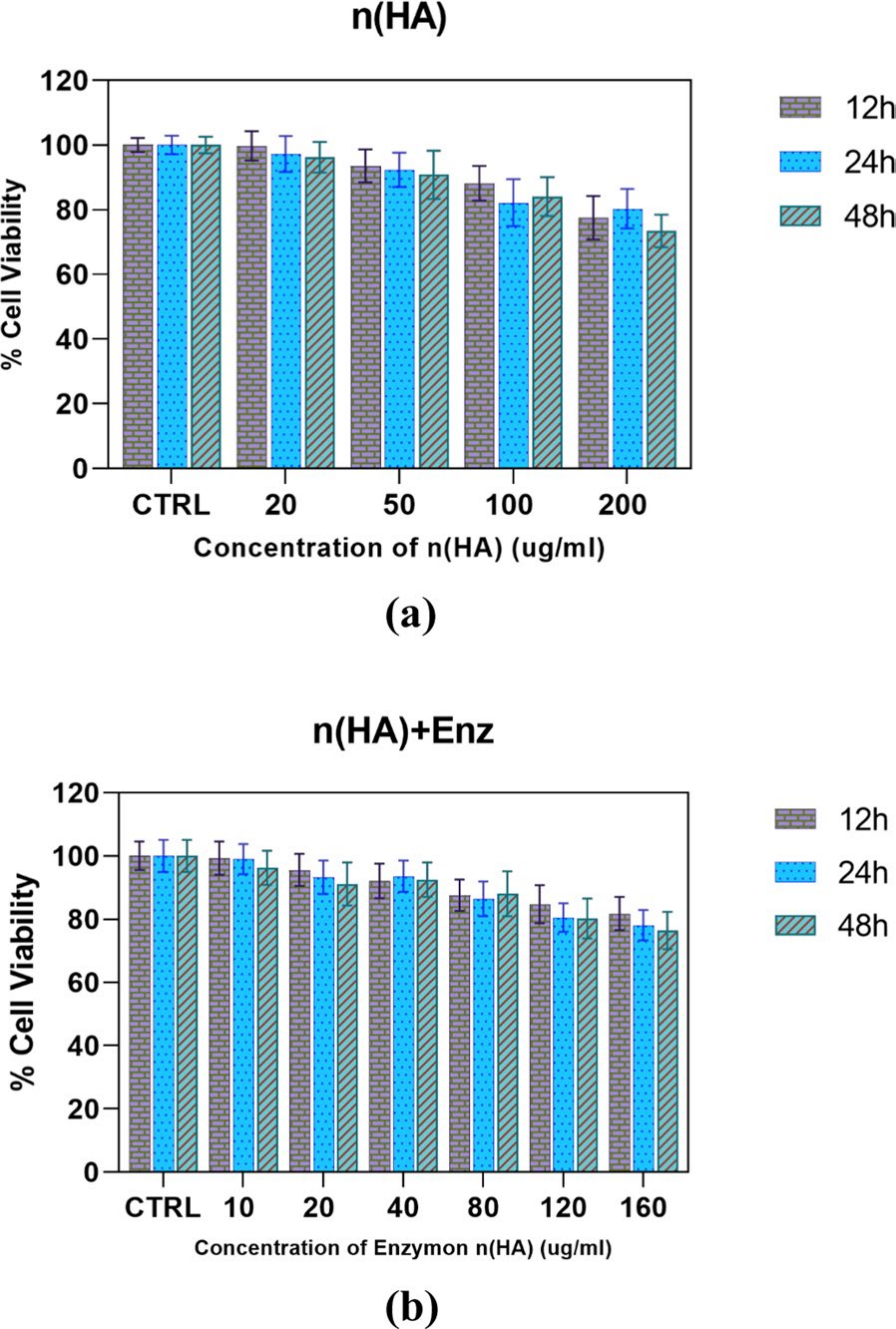
**a** Viability results of the test made using MTT assay on PC12 cells. Control is PC12 without a sample. Different concentrations of nano-hydroxyapatite. **b** Viability results of the test made using MTT assay on PC12 cells. Control is PC12 without a sample. Different concentrations of immobilized enzyme on nano-hydroxyapatite

For the purpose of examining cytotoxic effects, the MTT test was used. Several concentrations of the immobilized enzyme and nano-hydroxyapatite were examined. The findings of a 2017 investigation by Daneshjou et al. indicated that chondroitinase alone was not cytotoxic (Daneshjou et al. 2020). According to (Fig. 7), up to a concentration of 100 g/ml for a maximum of 24 h, hydroxyapatite nanoparticles do not exhibit any discernible toxicity. Moreover, there is no discernible toxicity for the immobilized enzyme on hydroxyapatite nanoparticles up to a concentration of 120 g/ml for a maximum of 24 h (Fig. 7). base on the aforementioned approach, the enzyme was immobilized and the main problem related to repeated injections was relatively resolved. therefore this nanosystem can be introdiuced for medical usage.

## Discussion

chABCI could be a promising molecule for the treatment of spinal cord injury, however, its maintained conveyance remains a challenge. There are common challenges of drug conveyance to the harmed spinal cord. The presence of the blood-spinal cord boundary avoids the dissemination of most particles from the blood to the spinal cord. Researchers have utilized fibrin, agarose, or chitosan gels to localized chABC at the location of SCI, be that as it may, these gels must be surgically embedded and within the case of agarose, require nearby cooling (Pakulsa 2016; Wilems et al. 2015; Hyatt et al. 2010; Lee et al. 2010). Also, the spinal cord is delicate to compressive constrain, so any drug conveyance vehicle must not swell or encroach upon the cord and there are challenges related to protein therapeutics. The maintained release is frequently accomplished by encapsulating proteins in PLGA particles that require cruel preparing conditions negative to protein structure and action (Pakulsa 2016; Van de Weert et al. 2000). chABCI is thermally unsteady and particularly troublesome to release for expanded periods (Pakulsa 2016; Tester et al. 2007). Efforts have been made to progress chABCI steadiness during release either utilizing adjuvants or by mutagenesis (Nazari-Robati et al. 2013). ChABCI was also secreted from lipid microtubules containing trehalose as a stabilizer (Pakulsa 2016; Wilems et al. 2015; Lee et al. 2010). There's as it were one report of effective chABCI encapsulation in poly(lactic acid) microspheres, in any case, the coming about microspheres are huge and unlikely to be injected through a fine needle (Pakulsa 2016; Huang et al. 2003). Also, porous silicon nanoparticles, chitosan-based scaffolds and PDLLA microspheres, magnetic Fe_3_O_4_ nanoparticles, and magnetic Fe_3_O_4_ nanoparticles coated with dextran have been used to immobilize the chABCI enzyme (Daneshjou et al. 2017; Askaripour et al. 2019; Huang et al. 2003; Askaripour et al. 2020). A perfect system for the distribution of chABCI to the harmed spinal cord would be biocompatible, negligibly intrusive, negligibly swelling, stay localized at the damaged location, provide chABCI over the time period of CSPG upregulation, and corrupt over time to hinder the requirement for surgical evacuation. Additionally, it must be manufactured under gentle conditions to protect chABCI activity. None of the chABC distribution systems listed above have all of these characteristics (Pakulsa 2016). The hydroxyapatite nanoparticles used in this study, with a diameter of 25 nm, can cross the blood barrier of the spinal cord and can be maintained for a long time, after injection, with a stable drug release. The loading of chABCI on hydroxyapatite nanoparticles does not require hard processing, and hydroxyapatite is a biocompatible material with the ability to guide bone growth, minimally stimulating the immune system, and minimally swelling, and it has been used in the treatment of bone infections before (Shuaishuai et al. 2023). Previous studies showed that the extraction and purification of the enzyme from Proteus vulgaris bacteria are done as an important source of the chABCI. ChABCI enzyme gene was previously cloned and expressed in E.coli bacteria (Nazari-Robati et al. 2012). The gene sequence used was consistent with the sequence reported by Ryan et al., which was consistent with the amino acid sequence reported by Huang et al. (Ryan et al. 1994; Huang et al. 2003). The expressed chABCI enzyme has a histidine sequence at the amino end, which enables its single-step, rapid and abundant purification through the formation of interactions with nickel ions in the chromatography column (Prabhakar et al. 2005). This method has already been used to purify AC, B, ABC Ι, and ABC ΙΙ chondroitinases (Prabhakar et al. 2005; Prabhakar et al. 2009). The objective of any drug conveyance system is to supply useful amounts of medicine to the right site within the body to attain it expeditiously and to preserve the required drug concentration (Ahuja et al. 2009). An ideal drug delivery system should be ineffective, biodegradable, highly biocompatible, and suitable for patient use (Ukmar et al. 2010). The advancement of immobilization methods for the chABC1 recommends a potential way to reuse it numerous times. In this study, the point was to capture the protein inside or on the surface of the insoluble material whereas keeping up its catalytic activity. With the plausibility of immobilizing the chABCI on distinctive substrates, there's still a challenge to discover a substrate that has the specified characteristics to immobilize the enzyme with high proficiency, and strength to resist distinctive prepare conditions at a competitive cost. The immobilization approach gives nonstop execution and progressed biocatalyst steadiness. Ordinarily, the support ought to have a high surface region to permit the immobilization of noteworthy amounts of proteins, hydrophilicity to guarantee great diffusivity of the substrate, and low dissolvability to elude product defilement (Coutinho et al. 2018). Combination therapy based on nanotechnology with nanocarriers such as dendrimers, liposomes, carbon nanotubes, polymeric drug compounds, micelles, and polymeric, ceramic, and inorganic nanoparticles has attracted much attention. Combined conveyance of drugs utilizing nanocarriers has the advantage of ensuring the drugs from debasement within the reticuloendothelial system, in this manner, empowering high concentrations of drugs at the target location with decreased poisonous impacts and with much lower dosages (Ram Prasad et al. 2020). The first part of the research included the expression and purification of the chABCI, the second part of the research included the immobilization of the chABCI on hydroxyapatite nanoparticles, and the third part included the characterization of the nanosystem containing the chABCI immobilized on hydroxyapatite nanoparticles. Then, the type of dominant interaction in the immobilization process of the chABCI was detected by Ft-IR analysis. The chABCI was immobilized on hydroxyapatite nanoparticles at three times of 1, 4, and 12 h at 4 °C and evaluated. The results show that coordination bonds are formed between Ca^2+^ sites of hydroxyapatite and COO− sites of amino acids. These results showed that the immobilization process is carried out by absorption by chelating the ions in the nano substrate with enzymatic amino acids. Immobilization was done with a simple, fast, and efficient method. The findings indicate that the enzyme maintained its activity for 4 h, but in 1 and 12-h immobilization, enzyme activity decreases and the nanosystem maintains its activity for about 1 month. The nanosystem was characterized by using field emission scanning electron microscopy, FT-IR spectroscopy, and X-ray diffraction (XRD). The FESEM image of hydroxyapatite nanoparticles, based on what was done in the case of immobilized dextranase on hydroxyapatite nanoparticles, seemed to cover a single layer of enzyme on the nanoparticles. Also, based on FESEM images of the immobilized dextranase on hydroxyapatite nanoparticles, the successful immobilization of the chABCI on hydroxyapatite nanoparticles was confirmed (Ding et al. 2020). A comparison of the IR spectra before and after chABCI immobilization showed that chABCI adsorption on the hydroxyapatite nanoparticles significantly changed the area and intensity of the peaks that appeared. The X-ray diffraction pattern of hydroxyapatite nanoparticles before and after immobilization of chABCI is similar to the diffraction pattern of hydroxyapatite nanoparticles before and after immobilization of dextranase (Ding et al. 2020). The stability of nanosystem (hydroxyapatite containing enzyme) increased compared to the free enzyme. In a study conducted by Askaripour et al., the stability of chABCI immobilized on Fe_3_O_4_ magnetic nanoparticles is significantly higher than the free form at low temperatures such as 4 °C. Also, in vitro release showed that ~ 94% of the enzyme is released within 6 h (Askaripour et al. 2019). Meanwhile, the release of immobilized chABCI on hydroxyapatite nanoparticles is very quick at first and then its speed decreases. Also, the results regarding the stability of the chABCI immobilized on porous silicon nanoparticles indicated that the immobilized enzyme at 4 °C is more stable than the free enzyme, and about 50% of the enzyme is released after 120 min. It decreases to 25% after 300 min and it remains constant for 1 day (Daneshjou et al. 2017). Askaripour et al. reported that the immobilized chABCI on Fe_3_O_4_ magnetic nanoparticles with dextran coating could maintain a higher percentage of its initial activity in comparison with the free enzyme at 4 °C. In addition, 70% of the chABCI is released after 9 h (Askaripour et al. 2020). While the release of enzyme from hydroxyapatite nanoparticles lasted for a duration of one week. The amount the nanosystem synthesized and used in this study was not toxic to PC12 cells. In a study conducted by Abbasi et al., it was shown that nano-hydroxyapatite particles at a concentration of 0.5 to 1 mg/ml in 24, 48, and 72 h had the highest cellular toxicity on human oral epithelial cells. Concentrations less than 0.05 mg/ml revealed the best biocompatibility, similar to Nano hydroxyapatite that exhibited the best biocompatibility at concentrations less than 0.05 mg/ml. From 2.5 to 5 mg/ml, the cellular toxicity of nano-hydroxyapatite particles decreased at all times (Abbasi et al. 2016). Finally, It can be summarized as follows:

Drug carriers are crucial components of the drug delivery system because they capture, hold onto, and release the medication gradually over time. Thus, features such as drug absorption and release, formulation stability, biocompatibility and biodistribution, and usefulness should be carefully considered when choosing carriers for drug administration. Researchers have suggested enzyme immobilization to get around the drawbacks of employing the pharmacological enzyme chABCI, which is short-lived. After damage, axon regeneration was aided by nanoparticles that released chABCI and protected the enzyme from quick deterioration. It is anticipated that hydroxyapatite nanoparticles would be investigated as a chABCI matrix due to their great effectiveness. In this work, we discovered that the chABCI can be effectively immobilized on hydroxyapatite using a fairly simple adsorption approach that creates a coordination link between the enzyme and the hydroxyapatite and is augmented by electrostatic interactions. When utilized as carriers for the transport of pharmaceuticals and other therapeutic agents, hydroxyapatite nanoparticles may enhance the bioavailability, predictable therapeutic response, high effectiveness, safety, and sustained and long-term release. Hydroxyapatite provides better-controlled medication release compared to other drug carriers, and it also has lower toxicity and adverse effects. The number of injections will be reduced thanks to the drug nanosystem including chABCI based on hydroxyapatite, which is projected to have a strong therapeutic impact on the breakdown of chondroitin sulfate proteoglycans (CSPGs) at the site of spinal cord damage. By monitoring the enzyme's daily activity, the stability of this nanosystem with an enzyme immobilized on hydroxyapatite nanoparticles was determined. According to the results, the enzyme maintained its activity during the first 4 h after stabilization but declined throughout the first and second 12 h. Also, the findings show that, in contrast to the free enzyme, this nanosystem becomes very stable at temperatures of 25 °C and 37 °C and maintains its activity for about a month. Hence, this nanosystem including chABCI and hydroxyapatite nanoparticles is regarded to address the drawback of employing free enzyme, which rapidly loses its activity, and is suitable for clinical use.

## Data Availability

All data generated or analyzed during this study are included in this published article [and its Additional files].

## References

[CR1] Abbasi F, Sattari M, Jalayer Naderi N, Soroushzad M (2016). Cytotoxicity of nano-hydroxyapatite on human-derived oral epithelium cell line: an in vitro study. Tehran Univ Med J.

[CR2] Ahuja G, Pathak K (2009). Porous carriers for controlled/modulated drug delivery. Indian J Pharm Sci.

[CR3] Askaripour H, Vossoughi M, Khajeh K, Alemzadeh I (2019). Magnetite nanoparticle as a support for stabilization of chondroitinase ABCI. Artif Cells Nanomed Biotechnol.

[CR4] Askaripour H, Vossoughi M, Khajeh K, Alemzadeh I (2020). Examination of chondroitinase ABC1 immobilization onto dextran-coated Fe_3_O_4_ nanoparticles and its in-vitro release. J Biotechnol.

[CR5] Bellucci D, Braccini S, Chiellini F, Balasubramanian P, Boccaccini AR, Cannillo V (2019). Bioactive glasses and glass-ceramics versus hydroxyapatite: comparison of angiogenic potential and biological responsiveness. J Biomed Mater Res.

[CR6] Bosio VE, Islan GA, Martínez YN, Durán N, Castro GR (2016). Nanodevices for the immobilization of the Busch S A, Silver J (2007) The role of extracellular matrix in CNS regeneration. Current Opinion in Neurobiology 17:120–127.rapeutic enzymes. Crit Rev Biotechnol.

[CR7] Bradbury EJ, Carter LM (2011). Manipulating the glial scar: chondroitinase ABC as a therapy for spinal cord injury. Brain Res Bull.

[CR8] Bradford MM (1976). A rapid and sensitive method for the quantitation of microgram quantities of protein utilizing the principle of protein-dye binding. Anal Biochem.

[CR9] Busch SA, Silver J (2007). The role of extracellular matrix in CNS regeneration. Curr Opin Neurobiol.

[CR10] Ch HI, Hsu SH, Chen MT, Hsieh CH, Ch KW, Cheng H, Huang Yi Y (2011). Controlled release of chondroitinase ABC1n chitosan-based scaffolds and PDLLA microspheres. Carbohyd Polym.

[CR11] Coutinho TC, Rojas MJ, Tardioli PW, Paris EC, Farinas CS (2018). Nanoimmobilization of β-glucosidase onto hydroxyapatite. Int J Biol Macromol.

[CR12] Coutinho TC, Tardioli PW, Farinas CS (2020). Phytase immobilization on hydroxyapatite nanoparticles improves its properties for use in animal feed. Appl Biochem Biotechnol.

[CR13] Daneshjou S, Dabirmanesh B, Rahimi F, Khajeh K (2017). Porous silicon nanoparticle as a stabilizing support for chondroitinase. Int J Biol Macromol.

[CR14] Daneshjou S, Dabirmanesh B, Rahimi F, Jabbari S, Khajeh K (2020). Catalytic parameters and thermal stability of chondroitinase ABCI on red porous silicon nanoparticles. J Biotechnol.

[CR15] Ding Y, Zhang H, Wang X, Zu H, Wang C, Dong D, Lyu M, Wang S (2020). Immobilization of dextranase on nano-hydroxyapatite as a recyclable catalyst. Materials.

[CR16] Faulkner JR, Herrmann JE, Woo MJ, Tansey KE, Doan NB, Sofroniew MV (2004). Reactive astrocytes protect tissue and preserve function after spinal cord injury. J Neurosci.

[CR17] Fawcett JW, Asher RA (1999). The glial scar and central nervous system repair. Brain Res Bull.

[CR18] Huang W, Lunin VV, Li Y, Suzuki S, Sugiura N, Miyazono H, Cygler M (2003). Crystal structure of *Proteus vulgaris* chondroitin sulfate ABC lyase Ι at 1. 9 A resolution. J Mol Biol.

[CR19] Hyatt AJT, Wang D, Kwok JC, Fawcett JW, Martin KR (2010). Controlled release of chondroitinase ABC from fibrin gel reduces the level of inhibitory glycosaminoglycan chains in the lesioned spinal cord. J Control Release.

[CR20] Konwarh R, Karak N, Rai SK, Mukherje AK (2009). Polymerassisted iron oxide magnetic nanoparticle immobilized keratinase. Nanotechnology.

[CR21] Kumar S, Sharma JG, Maji S, Malhotra BD (2016). A biocompatible serine functionalized nanostructured zirconia-based biosensing platform for non-invasive oral cancer detection. RSC Adv.

[CR22] Lee H, McKeon RJ, Bellamkonda RV (2010). Sustained delivery of thermostabilized chABC enhances axonal sprouting and functional recovery after spinal cord injury. Proc Natl Acad Sci.

[CR23] Mizushima Y, Ikoma T, Tanaka J, Hoshi K, Ishihara T, Ogawa Y, Ueno A (2006). Injectable porous hydroxyapatite microparticles as a new carrier for protein and lipophilic drugs. J Control Release.

[CR24] Moslemi D, Mortazavi S, Ghoreishi M (2015). Synthesis of nano hydroxyapatite: application in drug delivery of sulfasalazine. J Nanostruct.

[CR25] Muir E, De Winter F, Verhaagen J, Fawcett J (2019). Recent advances in the therapeutic uses of chondroitinase ABC. Exp Neurol.

[CR26] Naderi MS, Moghadam TT, Khajeh K, Ranjbar B (2018). Improving the stability of chondroitinase ABC I via interaction with gold nanorods. Int J Biol Macromol.

[CR27] Nazari-Robati M, Khajeh K, Aminian M, Fathi-Roudsari M, Golestani A (2012). Co-solvent mediated thermal stabilization of chondroitinase ABC1 from *Proteus vulgaris*. Int J Biol Macromol.

[CR28] Nazari-Robati M, Khajeh K, Aminian M, Mollania N, Golestani A (2013). Enhancement of thermal stability of chondroitinase ABC I by site-directed mutagenesis: an insight from Ramachandran plot. Biochem Biophys Acta.

[CR29] Nur Farahiyah M, Radzali O, Fei Y (2014). Nanoporous hydroxyapatite preparation methods for drug delivery applications. Rev Adv Mater Sci.

[CR30] Pakulska MM (2016) Combined delivery of chondroitinase ABC (ChABC) and stroma cell derived factor 1α (SDF 1α) for spinal cord regeneration. Dissertation, University Of Toronto

[CR31] Prabhakar V, Capila I, Bosques CJ, Pojasek K, Sasisekharan R (2005). Chondroitinase ABC Ι from Proteus vulgaris: cloning, recombinant expression, and active site identification. Biochem J.

[CR32] Prabhakar V, Capila I, Soundararajan V, Raman R, Sasisekharan R (2009). Recombinant expression, purification, and biochemical characterization of chondroitinase ABC ΙΙ from *Proteus vulgaris*. J Biol Chem.

[CR33] Prasanna APS, Venkatasubbu GD (2018). Sustained release of amoxicillin from hydroxyapatite nanocomposite for bone infections. Prog Biomater.

[CR34] Qi D, Min G, Li X, Jiangli L (2020). Immobilization of pectinase onto porous hydroxyapatite/calcium alginate composite beads for improved performance of recycle. ACS Omega.

[CR35] Ram Prasad S, Jayakrishnan A, Sampath Kumar TS (2020). Hydroxyapatite-dextran methacrylate core/shell hybrid nanocarriers for combinatorial drug therapy. J Mater Res.

[CR36] Ryan MJ, Khandke KM, Tilley BC, Lotvin JA (1994) Cloning and expression of the Chondroitinase I and II genes from *Proteus vulgaris*. Patent WO 94/25567

[CR37] Shuaishuai W, Tongtong Z, Dapeng W, Mingran Z, Xukai W, Yue Y, Hengliang D, Guangzhi W, Minglei Z (2023). Implantable biomedical materials for treatment of bone infection. Front Bioeng Biotechnol.

[CR38] Tester NJ, Plaas AH, Howland DR (2007). Effect of body temperature on chondroitinase ABC’s ability to cleave chondroitin sulfate glycosaminoglycans. J Neurosci Res.

[CR39] Ukmar T, Planinsek O (2010). Ordered mesoporous silicates as matrices for controlled release of drugs. Acta Pharm.

[CR40] Van de Weert M, Hennink WE, Jiskoot W (2000). Protein instability in poly(lactic-*co*-glycolic acid) microparticles. Pharm Res.

[CR41] Wen Y, Li J, Lin H, Huang H, Song K, Duan K, Weng J (2021). Improvement of drug-loading properties of hydroxyapatite particles using triethylamine as a capping agent: a novel approach. Crystals.

[CR42] Wilems TS, Sakiyama-Elbert SE (2015). Sustained dual drug delivery of anti-inhibitory molecules for the treatment of spinal cord injury. J Controlled Rel.

[CR43] Wu CC, Huang ST, Tseng TW, Rao QL, Lin HC (2010). FT-IR and XRD investigations on sintered fluoridated hydroxyapatite composites. J Mol Struct.

[CR44] Yamagata T, Saito H, Habuchi O, Suzuki S (1968). Purification and properties of bacterial chondroitinases and chondrosulfatases. J Biol Chem.

[CR45] Zdarta J, Budzinska K, Kolodziejczak-Radzimska A, Klapiszewski Ł, Siwinska-Stefanska K, Bartczak P, Piaasecki A, Maciejewski H, Jesionowski T (2015). Hydroxyapatite as a support in protease immobilization process. Physicochem Probl Min Process.

[CR46] Zuidema JM, Gilbert RJ, Osterhout DJ (2016). Nanoparticle technologies in the spinal cord. Cells Tissues Organs.

